# Comparison between the Correlations of Retinal Nerve Fiber Layer Thickness Measured by Spectral Domain Optical Coherence Tomography and Visual Field Defects in Standard Automated White-on-White Perimetry versus Pulsar Perimetry

**DOI:** 10.1155/2017/8014294

**Published:** 2017-10-08

**Authors:** Maged Alnawaiseh, Lisann Hömberg, Nicole Eter, Verena Prokosch

**Affiliations:** ^1^Department of Ophthalmology, University of Muenster Medical Center, Muenster, Germany; ^2^Department of Ophthalmology, University of Mainz, Mainz, Germany

## Abstract

**Purpose:**

To compare the structure-function relationships between retinal nerve fiber layer thickness (RNFLT) and visual field defects measured either by standard automated perimetry (SAP) or by Pulsar perimetry (PP).

**Materials and Methods:**

263 eyes of 143 patients were prospectively included. Depending on the RNFLT, patients were assigned to the glaucoma group (group A: RNFL score 3–6) or the control group (group B: RNFL score 0–2). Structure-function relationships between RNFLT and mean sensitivity (MS) measured by SAP and PP were analyzed.

**Results:**

Throughout the entire group, the MS assessed by PP and SAP correlated significantly with RNFLT in all sectors. In the glaucoma group, there was no significant difference between the correlations RNFL-SAP and RNFL-PP, whereas a significant difference was found in the control group.

**Conclusions:**

In the control group, the correlation between structure and function based on the PP data was significantly stronger than that based on SAP.

## 1. Introduction

Glaucoma is characterized by visual field defects and visual impairment due to retinal ganglion cell (RGC) loss and is one of the leading causes of blindness [1]. Evaluation of visual field defects is very important for monitoring glaucoma. White-on-white standard automated perimetry (SAP) is the “gold standard” for visual field testing, glaucoma follow-up, and identification of disease progression. However, SAP is highly influenced by patient attention levels and compliance and reveals functional defects only after substantial structural damage has already occurred [2, 3]. Modern perimetric tests, such as frequency-doubling technology (FDT) and Pulsar perimetry (PP), have been developed to provide early visual field (VF) defect detection [4, 5].

PP was developed by Gonzales-Hernandez and co-workers in 2000 [4] and can be used to test temporal and spatial contrast sensitivity functions simultaneously [6]. Various studies describe a higher sensitivity of Pulsar perimetry in comparison to SAP in detecting VF-loss in early glaucoma [6–8].

Moreover, combining the structure and function might help to detect glaucomatous changes earlier. Imaging of the retinal nerve fiber layer (RNFL) and the optic nerve head (ONH) using optical coherence tomography (OCT) is used to detect and monitor progression of glaucoma. Its correlation with different functional perimetry parameters has attracted increasing interest over the last few years [9–12].

This study aims to evaluate the structure-function relationship between RNFL thickness (RNFLT) measured by SD-OCT (Spectralis HRA-OCT (Heidelberg Engineering Inc., Heidelberg, Germany)) and retinal mean sensitivity (MS) measured by SAP or PP in glaucomatous patients and in a control group (healthy subjects, glaucoma suspects, and initial glaucoma cases). In addition, we compare for the first time the structure-function relationships of OCT and SAP versus OCT and PP in the different structural and functional sectors.

## 2. Materials and Methods

### 2.1. Subjects and Selection Criteria

This study included 263 eyes of 143 patients, consecutively enrolled between February 2014 and July 2014 at the Dept. of Ophthalmology, University of Muenster Medical Center. The study was approved by the Ethics Committee of the University of Muenster, North Rhine Westphalia, Germany. All subjects signed informed consent prior to any examination and the study followed the tenets of the Declaration of Helsinki. Inclusion criteria were a minimum best-corrected visual acuity of 0.6 and spherical equivalent within ±6 diopters (D). Patients with previous retinal surgery, macular or vitreoretinal disease, closure-angle glaucoma, neurological disease, diabetes, or dense cataract reducing RNFL measurements were excluded.

### 2.2. Examinations

#### 2.2.1. Visual Field Testing

All participants underwent visual field testing (SAP, PP). SAP was performed with the automated Humphrey Visual Field Analyzer II (HFA II, model 750; Carl Zeiss Meditec AG), using the standard program of the 30–2 Swedish interactive threshold algorithm (SITA fast) with a size III visible light stimulus, maximum intensity of 10,000 apostilbs (asb), and stimulus duration of 200 ms on a background illumination of 31.5 asb (10 cd/m^2^), or with the Octopus 600 analyzer (Haag-Streit AG, Schlieren, Switzerland). PP was performed with the Octopus 600, (Haag-Streit AG, Schlieren, Switzerland), using TOP strategy (tendency-orientated perimetry) with the 32 P distribution of points (similar to that used with the 30–2 SITA) with a maximum intensity of 200 apostilbs (asb) and with a stimulus duration of 500 ms on a background illumination of 100 asb (31.8 cd/m^2^). Refractive errors were corrected according to the manufacturer's recommendations. Though most participants were familiar with visual field testing (SAP), instructions on how to perform the visual field test were given before any testing. Fixation was controlled by the examiner during the test. A resting period of 10 minutes was offered between tests and no specific test order was followed. Reliability indices for SAP and Pulsar perimetry were set to false positive, false negative, and fixation loss errors of less than 30%.

#### 2.2.2. Measurement of RNFL Thickness

OCT was performed with spectral-domain OCT (Spectralis; Heidelberg Engineering, Heidelberg, Germany) by an expert examiner. RNFL thickness was measured around the optic nerve head. The Spectralis OCT software, Heidelberg Explorer (HEE, version 5.3; Heidelberg Engineering Co., Heidelberg, Germany), was used for the automatic segmentation of the RNFL and for calculation of RNFLT. Upper and lower boundary lines were artificially and automatically adjusted when misplaced. Each sector was colored yellow or red if the RNFL measurement fell outside the lower 95% and 99% of the centile ranges, respectively.

RNFL thickness deviation maps were assigned to different groups, as described by Leung et al. [13, 14]. In the present study, eyes of patients with a score of 0–2 (healthy subjects, glaucoma suspects, and initial glaucoma cases) were assigned to group B, while patients with a score of 3–6 were assigned to group A (glaucoma group). Subjects were assigned to group B (healthy subjects, glaucoma suspects, and initial glaucoma cases) or group A (glaucoma group) based on RNFLT defects with reference to the Spectralis SD-OCT RNFL thickness deviation map, without regard to perimetry findings. Thus, the control group is not strictly normal, but a heterogeneous group of normal and suspect subjects.

**Figure 1:**
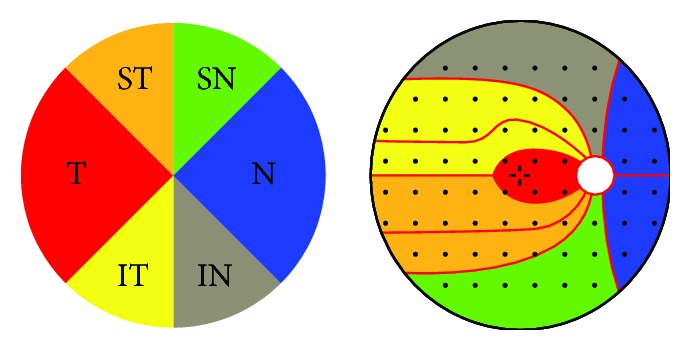
Retinal nerve fiber layer sectors and Octopus visual field clusters. IN: inferonasal; IT: inferotemporal; SN: superonasal; ST: superotemporal; T: temporal; L: nasal. The map is based on the structure-function map developed by Garway-Heath and colleagues [15].

Each visual field was subdivided according to the structure-function map developed by Garway-Heath et al. into six sectors [15]; the sectors were named according to their structural location on the ONH (Figure 1). MS values were calculated for each visual field cluster and correlated with the RNFLT of the corresponding sector.

#### 2.3. Data Analysis and Statistics

Data management was performed with Microsoft Excel 2013. We used IBM SPSS® Statistics 22 for Windows (IBM Corporation, Somers, NY, USA) for statistical analyses. As the data was not normally distributed, the degree of correlation between two variables (RNFL and SAP/PP) was expressed as Spearman's correlation coefficient, assuming the left and right eyes of the same patient to be independent. Structure-function correlation coefficients (RNFL-SAP and RNFL-PP) were compared using a statistical procedure for comparing dependent correlations, as described by Field [16]. The level of statistical significance was set at *p* ≤ 0.05. Data are presented as mean ± standard deviation.

## 3. Results

In this study, 263 eyes of 143 patients (70 eyes in group A (RNFL score of 3–6) and 193 eyes in group B (RNFL score 0–2)) were enrolled between February 2014 and July 2014 in our department. Mean patient age was 46 ± 21 years (range: 12–91 years).  Table 1 summarizes the characteristics of the study groups (Table 1).

In the entire group, the structure-function relationship between RNFL thickness (RNFLT) and retinal mean sensitivity (MS) as measured by SAP and PP showed significant correlations in all sectors (RNFLT and SAP: *p* < 0.001 in all sectors except for the nasal sector (*p* = 0.003)) (RNFLT and PP: *p* < 0.001 in all sectors). The highest *r* value in the entire group was seen between RNFLT and PP for global thickness (Table 2).

The strongest structure-function correlation (for both SAP and PP) was found in group A (Table 3). There was a significant correlation in all sectors except for the nasal sector. The highest *r* values for SAP and PP were found in the superotemporal and inferotemporal sectors (SAP superotemporal: *r* = 0.74, *p* < 0.001; inferotemporal: *r* = 0.75, *p* < 0.001; PP superotemporal: *r* = 0.72, *p* < 0.001; inferotemporal: *r* = 0.73, *p* < 0.001) (Table 3).

Interestingly, in group B, there was no significant correlation between RNFLT and SAP in any sector, while the global RNFL thickness and the RNFLT in the inferior-temporal, superior-temporal, and superior-nasal sectors correlated with the MS of the corresponding cluster on PP (global RNFL thickness: *r* = 0.31, *p* < 0.001; inferior-temporal sector: *r* = 0.31, *p* < 0.001; superior-temporal sector: *r* = 0.30, *p* < 0,001; superior-nasal sector: *r* = 0.28, *p* < 0.001) (Table 4). A statistically significant difference between the Spearman's correlation coefficients of the RNFL-PP and RNFL-SAP was found for global RNFL thickness and the RNFLT in the inferior-temporal, superior-temporal, and superior-nasal sectors (Table 4).

## 4. Discussion

In this study, we investigated the structure-function relationship between the RNFLT of 6 sectors measured with SD-OCT and the MS of 6 corresponding PP and SAP visual field sectors. To our knowledge, this is the first study in which the structure-function relationship for Pulsar perimetry has been investigated and compared with the structure-function relationship for SAP.

In the programs for Octopus perimetry, the positions of test points are based on the nerve fiber bundles and the distance from the optic nerve head (ONH) [12]. This makes it easier to group functionally related test points into clusters and to couple these visual field clusters to defined areas of the ONH [12, 17]. In a recent study, Monsalve et al. [18] presented a structure-function map using OCT sectors and Octopus VF regions obtained completely from objective analysis of healthy participants and patients with glaucoma. This map shows reasonable agreement with that of Garway-Heath et al. [15, 18].

In the present study, the correlation was based on the structure-function correspondence map proposed by Garway-Heath et al. [15].

Naghizadeh et al. investigated the structure-function relationship between the MS values of 16 Octopus visual field clusters and RNFL thickness of the corresponding 16 peripapillary sectors (RTVue OCT). In this study, a significant correlation was found in all tested sectors, with the highest *R*^2^ (coefficient of determination) values occurring in the temporal, superotemporal, and inferotemporal RNFLT sectors. Naghizadeh et al. showed here that the visual field clusters of the Octopus G program can be applied for further detailed structure-function research. This map also corresponds reasonably well with the map described by Garway-Heath et al. [12, 16]. In contrast to the study of Naghizadeh et al., which investigated the structure-function relationship for 16 clusters of the Octopus perimetry [12], we have used the simplified clustering (6 clusters) as described by Garway-Heath et al. and used in other different studies [16, 18, 19], to allow correlation with the RNFL sectors of the Heidelberg Engineering OCT, and a structured comparison of the SAP findings of our study with other studies in the literature.

The strongest correlation between RNFLT and MS on SAP in our entire study group was seen in the superotemporal and inferotemporal sectors, and the correlation was significant in all sectors. Comparable results have been described in different studies for the analysis of the structure-function relationship between RNFLT and SAP [10, 20]. In this context, it is important to mention that the strength of correlation between MS and RNFLT varies between different studies, depending on the instruments used, patient factors or study population, the number of clusters used, and study design. All these factors can influence the results and make direct comparisons very difficult, so that small differences should be interpreted with caution.

For the SAP and Pulsar perimetry, the *r* values were highest in group A, and the correlation was significant in all sectors except the nasal sector. This was to be expected, because the greater the visual field damage, the stronger the correlations between OCT and SAP will be [11]. In group A, the highest correlation can be seen in the superior-temporal and inferior-temporal sectors, and comparable results have been described in other studies [10, 12]. The *r* values for SAP and PP in group A were similar and no significant difference could be found between the correlations OCT-SAP and OCT-PP in the glaucoma group. Both SAP and Pulsar perimetry are able to detect visual field loss in patients with advanced glaucoma and this VF loss correlates with RNFL thinning.

The correlation between MS on SAP and RNFLT in group B was not significant, and similar findings have been reported for the correlation between MS on SAP and RNFLT in patients with early glaucoma or suspected glaucoma [10, 11, 21].

The data for correlation of RNFLT to PP in group B are of particular interest. A significant correlation was found for global thickness and in three sectors. The strongest correlation was in the superior-temporal and inferior-temporal sectors and this was in line with our expectations and the fact that glaucomatous damage preferentially affects the superotemporal and inferotemporal areas of the ONH [22].

Pulsar perimetry was specifically designed for better detection of glaucoma [23], and when used alone or in combination with other functional and structural tests, it has shown a higher sensitivity than SAP in the diagnosis of early glaucoma [6]. SD-OCT has also been described as a helpful diagnostic tool for early detection of glaucoma [9, 21]. On the other hand, SAP is able to detect functional defects only after substantial structural damage has already occurred [2, 3]. These facts may explain the significant difference between the correlations RNFLT-SAP and RNFLT-PP.

Group B (control group) is a heterogeneous group of normal and suspect subjects according to the RNFL thickness classification. Hence, we might raise the following interpretation of the results: Pulsar would be able to identify changes or defects in the RNFLT in very initial phases of the disease, detecting changes in subjects right in the transition between normality and pathology, which SAP is not able to detect. This interpretation coincides with the reasons behind Pulsar's design and confirms the hypothesis that based its development.

This study has some limitations. First, patients were classified according to the RNFLT classification categories without consideration of VF parameters. However, this study was designed to find out the value of PP especially in this population and compare it with the SAP. Second, both Humphrey and Octopus perimeters, which are different in several technical aspects, were used in this study. Third, reliability criteria might be a bit relaxed in this study. Future research considering these limitations would be helpful to validate these findings.

In conclusion, in the glaucoma group, no notable differences were found in the strength of the structure-function relationships of RNFLT to PP and SAP. The strongest correlations between structure and function were seen mainly in the superotemporal and inferotemporal sectors of the ONH. In group B, the correlations were significant only for PP, and the correlation between structure and function is significantly stronger with PP than with SAP.

## Figures and Tables

**Table 1:** Characteristics of the study groups.

	Entire group (*n* = 263)	Group A (*n* = 70)	Group B (*n* = 193)	*p* val.
Age	44.92 ± 20.65	40.06 ± 20.22	58.30 ± 15.33	**<0.001**
Sex (m : w)	102 : 161	73 : 120	29 : 41	
RNFLT	88.37 ± 15.39	95.83 ± 8.10	67.79 ± 11.45	**<0.001**
MD (SAP)	−2.26 ± 4.03	−1.04 ± 2.18	−5.65 ± 5.7	**<0.001**
PSD/sLV (SAP)	2.89 ± 2.78	1.97 ± 0.97	5.42 ± 4.23	**<0.001**
MD (PP)^∗^	1.59 ± 3.58	0.51 ± 2.29	4.56 ± 4.67	**<0.001**
PSD/sLV (PP)	2.22 ± 1.24	1.82 ± 0.72	3.33 ± 1.63	**<0.001**

Bold: statistically significant differences between group A and group B. ^∗^Octopus instruments show defects with a positive MD sign.

**Table 2:** Structure-function relationship between RNFL thickness, measured with SD-OCT, and Pulsar perimetry or standard automated perimetry for the entire study group.

Entire group (*n* = 263)		Global thickness^∗^	Temporal inferior thickness^∗^	Temporal thickness	Temporal superior thickness^∗^	Nasal superior thickness^∗^	Nasal thickness^∗^	Nasal inferior thickness^∗^
RNFL and Pulsar	*r*	0.59	0.57	0.26	0.55	0.46	0.33	0.33
	*p*	**<0.001**	**<0.001**	**<0.001**	**<0.001**	**<0.001**	**<0.001**	**<0.001**
RNFL and SAP	*r*	0.38	0.40	0.27	0.40	0.24	0.18	0.21
	*p*	**<0.001**	**<0.001**	**<0.001**	**<0.001**	**<0.001**	**0.003**	**<0.001**

*r* Spe: Spearman's correlation coefficient. ^∗^Statistically significant difference between correlations for PP and SAP.

**Table 3:** Structure-function relationship between RNFL thickness, measured with SD-OCT, and Pulsar perimetry or standard automated perimetry for the glaucoma group (A).

Group A (70)		Global thickness^∗^	Temporal inferior thickness^∗^	Temporal thickness	Temporal superior thickness^∗^	Nasal superior thickness^∗^	Nasal thickness^∗^	Nasal inferior thickness^∗^
RNFL and Pulsar	*r*	0.56	0.73	0.43	0.72	0.29	0.08	0.26
	*p*	**<0.001**	**<0.001**	**<0.001**	**<0.001**	**0.016**	0.506	**0.033**
RNFL and SAP	*r*	0.60	0.75	0.49	0.74	0.39	0.08	0.34
	*p*	**<0.001**	**<0.001**	**<0.001**	**<0.001**	**0.001**	0.515	**0.004**

*r* Spe: Spearman's correlation coefficient. ^∗^Statistically significant difference between correlations for PP and SAP.

**Table 4:** Structure-function relationship between RNFL thickness, measured with SD-OCT, and Pulsar perimetry or standard automated perimetry for group B.

Group B (*n* = 193)		Global thickness^∗^	Temporal inferior thickness^∗^	Temporal thickness	Temporal superior thickness^∗^	Nasal superior thickness^∗^	Nasal thickness^∗^	Nasal inferior thickness^∗^
RNFL and Pulsar	*r* Spe	0.31	0.31	−0.03	0.30	0.28	0.05	−0.02
	*p* val.	**<0.001**	**<0.001**	0.722	**<0.001**	**<0.001**	0.537	0.794
RNFL and SAP	*r* Spe	0.06	0.13	0.04	0.12	0.05	−0.01	−0.10
	*p* val.	0.436	0.067	0.57	0.106	0.514	0.929	0.178

*r* Spe: Spearman's correlation coefficient. ^∗^Statistically significant difference between correlations for PP and SAP.
